# Relationship between the location of the popliteal artery and the tibial osteotomy plane in patients with medial and lateral unicompartmental knee arthroplasty: A retrospective analysis of preoperative magnetic resonance imaging and intraoperative findings

**DOI:** 10.1051/sicotj/2024058

**Published:** 2025-01-13

**Authors:** Tatsuya Kubo, Tsuneari Takahashi, Yuya Kimura, Takashi Ajiki, Eri Yasuda, Katsushi Takeshita

**Affiliations:** 1 Department of Orthopaedic Surgery, Shin Oyama City Hospital Oyama Japan; 2 Department of Orthopaedics, School of Medicine, Jichi Medical University Shimotsuke Japan; 3 Department of Orthopaedic Surgery, Nasu Chuo Hospital Otawara Japan; 4 Department of Orthopaedic Surgery, Ishibashi General Hospital Shimotsuke Japan

**Keywords:** Knee osteoarthritis, Popliteal artery, Unicompartmental knee arthroplasty, Vascular injury

## Abstract

*Purpose*: To clarify the location of the popliteal artery (PA) is relative to the tibial osteotomy plane in patients with medial and lateral unicompartmental knee osteoarthritis (KOA) undergoing UKA. *Methods*: Preoperative MRI and postoperative radiographs obtained from 50 patients with unicompartmental KOA who underwent fixed-bearing UKA were analyzed. The amount of tibial resection was determined from the surgical records, and a line was drawn parallel to the tibial posterior tilt angle on the sagittal MR image to create a virtual tibial cut line. The tibial resection width measured from the anteroposterior image of the postoperative radiograph was projected onto the transverse plane containing the intersection between the virtual tibial cut line and the posterior tibial cortex, after which a line was drawn parallel to the medial or lateral intercondylar ridge. We then determined whether the PA was within an extension of the osteotomy area. The shortest distance (Distance 1) between the posterior tibial cortex and the PA within the osteotomy area was measured. In addition, the shortest distance between the line extending the osteotomy posteriorly and the PA was measured (Distance 2). *Results*: The medial UKA (group M) and lateral UKA (group L) group comprised 41 and 9 cases. The percentage of PA located behind the osteotomy plane was significantly higher in group L than in group M [6/9 knees (66.7%) vs. 2/41 knees (4.9%); *P* < 0.001]. The distance 1 was 12.6 (4.3) mm in group M and 7.9 (3.7) mm in group L (*P* = 0.004). The distance2 was 11.1 (4.9) mm in group M and 2.6 (3.5) mm in group L (*P* < 0.001). *Conclusion*: During lateral UKA, the PA was often located behind the tibial osteotomy plane and close to the posterior tibial cortex. Nearly 5% of medial UKAs, the artery was located behind the osteotomy plane. *Level of Evidence*: Retrospective comparative LEVEL III study.

## Introduction

Knee osteoarthritis (KOA) is a degenerative joint disease characterized by the progressive erosion of articular cartilage and is one of the most common musculoskeletal diseases affecting 25,000,000 Japanese patients aged > 40 years who have radiographic KOA [1]. Total knee arthroplasty (TKA) is a widely accepted surgical procedure for the treatment of end-stage KOA that significantly improves pain relief and functional restoration [2]. Neurovascular complications rarely occur following TKA, with an incidence rate between 0.02% and 0.05%, which may be underestimated [3, 4]. Although vascular complications rarely occur after TKA, several cases of pseudoaneurysms affecting the genicular and popliteal vessels have been reported. In addition, arterial injury can be a severe complication that could necessitate amputation of the affected limb [5].

Unicompartmental knee arthroplasty (UKA) is another excellent surgical option for restoring painless function in patients with osteoarthritis of one knee compartment [6]. Although some complications have been reported, vascular injuries have not been well recognized [7–10], with no studies having described these serious problems to date. This study aimed to clarify the location of the popliteal artery (PA) relative to the tibial osteotomy plane in patients with medial and lateral unicompartmental KOA undergoing UKA.

## Methods

This retrospective study has included 50 KOA patients with unicompartmental KOA who underwent MOTO-UKA (Medacta, Castel San Pietro, Switzerland) between January 2023 and June 2024. An experienced knee surgeon comprehensively evaluated symptoms (e.g., pain and decline of activities of daily living), physical findings, and imaging findings (e.g., Kellgren–Lawrence (KL) grading scale [11] and knee osteoarthritis grading scale [12]). Surgical indications were KL grade 2, 3, and 4. Patients who had not responded to conservative treatment and expressed a desire to undergo UKA proceeded with surgical intervention. The exclusion criteria were previous knee arthroplasty or ligament reconstruction owing to the confounding effects of prior surgery on soft tissue around the knee. Sex, preoperative hip–knee angle (HKA; positive value indicates varus), and body mass index (BMI) were also evaluated.

### Radiological evaluation of PA location using magnetic resonance imaging (MRI)

**Figure 1:**
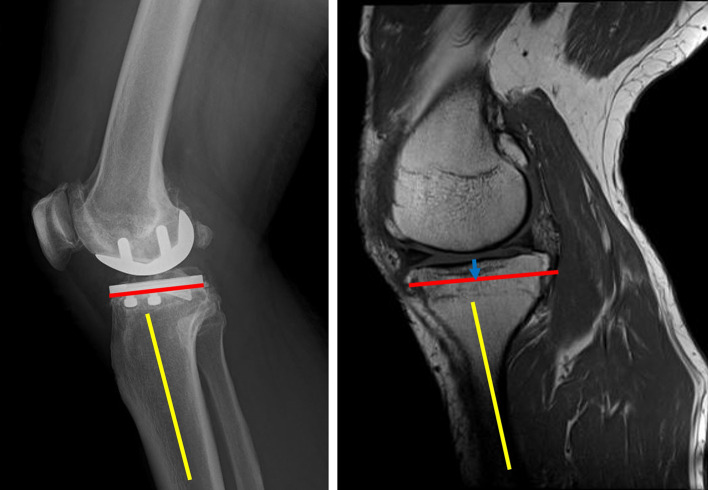
The tibial posterior tilt angle was measured from the postoperative lateral radiograph image. The amount of tibial resection was determined from the surgical record (blue arrow), and a line was drawn parallel to the tibial posterior tilt angle on the sagittal MRI image to create a virtual tibial cut line (red line).

**Figure 2:**
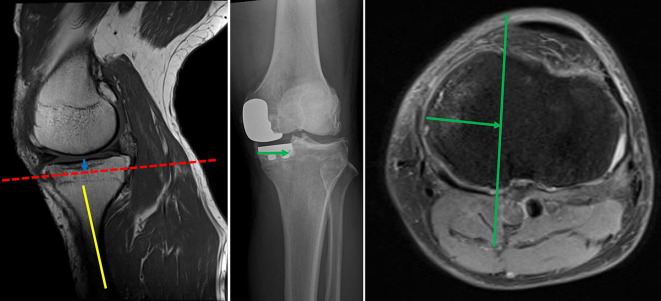
The tibial resection width (green arrow) measured from the anteroposterior image of the postoperative radiograph was projected onto the transverse plane containing the intersection of the virtual tibial cut line and the posterior tibial cortex, after which a line was drawn parallel to the medial or lateral intercondylar ridge (green line).

**Figure 3:**
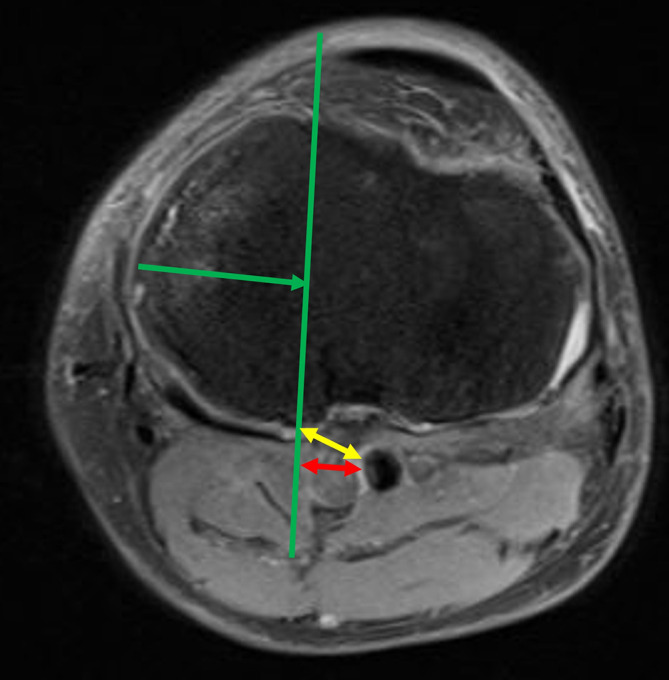
We determined whether the PA was within an extension of the osteotomy area. The shortest distance (yellow arrow) between the posterior tibial cortex and PA within the osteotomy area was measured (Distance 1). Moreover, the shortest distance between the line extending the osteotomy posteriorly and the PA was measured (Distance 2; red arrow). The distance 2 was determined to be 0 mm if the PA was within the extension of the osteotomy area.

In patients with knee osteoarthritis who visited the outpatient clinic for knee problems, if conservative treatment was ineffective and UKA was considered, all patients underwent MRI examination using a 3-T MRI system to determine whether UKA was appropriate (Siemens, Echelon, Germany). MRI was performed with the knee in extension, and coronal, sagittal, and axial images were obtained [13–15]. Full extension of the knee was maintained in the standard surface coil using an appropriately sized pillow to prevent knee flexion. Based on a previous study, the sagittal and coronal plane images were obtained using the following protocol to investigate the location of the PA with the knees in the extended position through MRI and proton density-weighted images with fat suppression. Notably, each section was 5-mm thick with a 0.5-mm intervening spacing and had a 512 × 512 matrix [14]. All MRIs were read by two board certified orthopedic surgeons specializing in knee surgery who participated in the study. MRI was performed not only to investigate the location of the PA but also for routine preoperative evaluation before UKA. The tibial posterior tilt angle was measured from the postoperative lateral short-film radiograph image. The line connecting the midpoints of the anterior-posterior width at the proximal tibia of 5 cm and 10 cm was used as the tibial axis, and the slope of the osteotomy surface was measured. The amount of tibial resection (blue arrow) was determined from the surgical record, we measured the resected tibial height during surgery with precise calipers and recorded it while taking into account bone loss due to saw cutting, and a line was drawn parallel to the tibial posterior tilt angle on the sagittal MR image to create a virtual tibial cut line (red line) (Figure 1). The tibial resection width (green arrow) measured from the anteroposterior image of the postoperative radiograph was projected onto the transverse plane containing the intersection of the virtual tibial cut line and the posterior tibial cortex, after which a line was drawn parallel to the medial or lateral intercondylar ridge of the tibia (green line) (Figure 2). We then determined whether the PA was within an extension of the osteotomy area. The shortest distance (Distance 1: yellow arrow) between the posterior tibial cortex and the PA within the osteotomy area was measured. In addition, the shortest distance between the line extending the osteotomy posteriorly and the PA was measured (Distance 2: red arrow) (Figure 3). Distance 2 was set to 0 mm if the PA was within the extension of the osteotomy area.

### Statistical analysis

The mean difference in the distance from the posterior cortex of the tibia to the PA between patients who underwent medial UKA (group M) and lateral UKA (group L) was determined. All data were presented as mean (standard deviation). Groups M and L consisted of 41 and 9 cases, respectively. A post hoc power analysis was performed using G*Power v.3.1 (Franz Faul, Kiel, Germany) [16]. Based on the sample size in each group, an alpha error of 0.05, a power of 0.8, the effect size was calculated to be 1.1. A *P*-value of less than 0.05 was deemed statistically significant, and statistical analysis was conducted using EZR software (R Commander, customised for medical statistics by Kanda et al., Japan) [17]. For comparison of means of continuous variables, *t*-tests were performed, and for comparison of categorical variables between the two groups, Fisher’s exact test was performed.

## Results

Patient characteristics are summarized in Table 1. The 50 patients included herein, 32 (74.4%) of whom were female, had a mean age of 72.7 (6.3) years and a mean BMI of 24.4 (3.3) in group M and 23.3 (4.4) kg/m^2^ in group L. Preoperative HKA was 4.3° (3.3°) in group M and 0.7° (2.9°) in group L. KL grade 2 was observed in 10 (24.4%) and 2 knees (22.2%) from groups M and L, respectively; KL grade 3 was observed in 20 (48.8%) and 5 knees (55.6%) from groups M and L, respectively; and KL grade 4 was observed in 11 (26.8%) and 2 knees (22.2%) from groups M and L, respectively. The percentage of PA located behind the osteotomy plane was significantly higher in group L than in group M [6/9 knees (66.7%) and 2/41 knees (4.9%), respectively; *P* < 0.001]. The Distance 1 was 12.6 (4.3) mm in group M and 7.9 (3.7) mm in group L, with the distance being significantly shorter in the latter (*P* = 0.004). The distance 2 was 11.1 (4.9) mm in group M and 2.6 (3.5) mm in group L, with the distance being significantly shorter in the latter (*P* < 0.001; Table 2).

**Table 1:** Demographic data of the included patients.

Characteristics	Group M (*n* = 41)	Group L (*n* = 9)	*P* value
Mean age (years)*	73.4 (5.2)	69.3 (10.0)	0.09
BMI (kg/m^2^)*	24.4 (3.3)	23.3 (4.4)	0.41
Male**	12 (29.2%)	1 (11.1%)	0.41
Female**	29 (70.7%)	8 (88.9%)	
HKA*	4.3 (3.4)°	0.7 (2.9)°	0.01
KL grade 2**	10 (24.4%)	2 (22.2%)	1.0
KL grade 3**	20 (48.8%)	5 (55.6%)	
KL grade 4**	11 (26.8%)	2 (22.2%)	

Data are expressed as mean (standard deviation).

* Comparison between groups using *t*-test.

** Comparison between groups using Fisher’s exact test.

NS, not significant; BMI, body mass index; HKA, Hip–Knee–Ankle angle; KL, Kellgren–Lawrence classification.

**Table 2:** Evaluation of the positional relationships between the popliteal artery and tibial cut plane.

	Group M	Group L	P value
Percentage of PA located behind the osteotomy plane*	2/41 knees (4.9%)	6/9 knees (66.7%)	*P* < 0.001
The Distance 1**	12.6 (4.3) mm	7.9 (3.7) mm	*P* = 0.004
The Distance 2**	11.1 (4.9) mm	2.6 (3.5) mm	*P* < 0.001

Data are expressed as mean (standard deviation).

* Comparison between groups using Fisher’s exact test.

** Comparison between groups using *t*-test.

PA, popliteal artery.

The Distance 1, the shortest distance between the posterior tibial cortex and the PA within the osteotomy area.

The Distance 2, the shortest distance between the line extending the osteotomy posteriorly and the PA.

## Discussion

This has been the first study published in English to clarify the effects of operated side (e.g., medial or lateral) on the distance from the posterior cortex of the proximal tibia to the anterior wall of the PA at the height of the tibial cut line during UKA. The most important finding of this study was that a high proportion of PAs were located behind the tibial osteotomy during lateral UKA. Although this was clearly expected anatomically, no study has yet objectively described this phenomenon as a precaution during lateral UKA, which is important in order to shed light on the risk for injury. Another important finding was that in medial UKA, the PA was located posterior to the tibial osteotomy in 5% of cases. Although it was generally considered that the PA was more lateral than the PCL, our findings suggested that the surgical saw should not be inserted deeper during tibial osteotomy, even in medial UKA. Although MRI is not routinely performed prior to UKA [14], the current study alerts surgeons to the immediate proximity of the artery to the surgical saw when performing the tibial cut. To prevent damage to the PA, it is important to adequately expose the joint and insert necessary retractors to protect the PA during surgery. In addition, we found that during UKA, the anterior wall of the PA was close to the posterior cortex of the proximal tibia at the site of osteotomy. Yoo and Chang studied healthy Korean volunteers and reported an artery-tibia distance of 2.7 mm in extension and 7.2 mm in 90° flexion, and it is assumed that keeping the knee in the flexed position when performing UKA is effective in preventing vascular injury [13]. Injuries to the PA during TKA surgery have been previously reported, with a postoperative incidence of 0.03%–0.23% [18–20]. Moreover, peripheral artery disease and revision arthroplasty have been identified as risk factors for knee PA injury [18, 21]. Unfortunately, current evidence on vascular injuries after UKA surgery has been scarce, with only a few cases of pseudoaneurysms having been reported [9, 10]. However, this does not indicate the absence of risk for vascular injury during UKA. In fact, one study showed that among all knee arthroplasty procedures performed in the US 1.05% in 2016 and 3.03% in 2019 were UKA surgeries, suggesting that the low incidence of vascular injury could be due to the small number of surgeries [22]. In addition, reports have shown that the number of surgeries for lateral UKA accounted for around 5%–10% of the number of surgeries for medial UKA [23, 24], which may contribute to the low number of reported PA injuries during UKA. In recent years, UKA has attracted considerable attention for its fast postoperative recovery and cost, promoting an increase in the number of surgeries [22, 25]. One can reasonably expect an increase in the reporting of PA injuries in the future, highlighting the importance of the findings presented herein, particularly in alerting surgeons to the risk for injury.

### Limitations

Several limitations to this study warrant discussion. First, distance measurements were not blinded given that the PACS system was used. Second, MRI was performed with the knees in the extended position only. Although measuring the artery-tibia distance in extension may underestimate the distance because the PA is thought to move more posteriorly in flexion than in extension, Zaidi et al. reported that there was no significant difference in the artery-tibia distance at the knee joint in extension and 90° flexion, and in some cases the PA is closer to the posterior tibia in extension than in flexion [26]. The elucidation of the relationships between the PA and the tibia in both the extended and flexed positions would enhance the value of our study. Although the present study exclusively examined the positional relationship in the extension position, measuring the distance in extension may offer some insight. This is because the PA may be closer proximity to the tibia in the extension position than in the flexion position. Third, it was not within the scope of this study to evaluate whether the axis of rotation of the tibial osteotomy referenced on the preoperative MRI was reproduced intraoperatively and postoperatively. Fourth, considering that there was no noticeable discrepancy between the width of the implant used and the measured tibial resection width, it was concluded that the magnification error of the anteroposterior radiographs used to assess the tibial resection width was not significant. Fifth, only patients who had undergone fixed-bearing UKA were evaluated. Therefore, our results cannot be generalized to patients who underwent mobile-bearing UKA, which we intend to clarify in the near future. Sixth, all patients included herein were Japanese; hence, our results cannot be considered universal and cannot be applicable to all ethnic groups. Finally, although our study compared medial UKA with lateral regarding the distance of the popliteal artery to the posterior tibial cortex and had sufficient power, the descriptive value of our findings is limited by the inclusion of only nine cases of lateral UKA.

## Conclusion

During lateral UKA, the PA was often located behind the tibial osteotomy surface and is close to the posterior tibial cortex. In 5% of medial UKAs, the artery was located behind the osteotomy plane. Our findings highlight the need for surgeons performing UKAs to be vigilant of the possibility of PA injury.

## Data Availability

Data and materials of this study are available from the corresponding author upon reasonable request.
